# Utilization of addiction treatment among U.S. adults with history of incarceration and substance use disorders

**DOI:** 10.1186/s13722-019-0138-4

**Published:** 2019-03-05

**Authors:** Jack Tsai, Xian Gu

**Affiliations:** 1 Department of Veterans Affairs (VA), New England Mental Illness Research, Education, and Clinical Center (MIRECC), 950 Campbell Ave., 151D, West Haven, CT 06516 USA; 2 Department of Psychiatry, Yale University School of Medicine, 300 George St., New Haven, CT 06511 USA; 3 Department of Biostatistics, Yale School of Public Health, 60 College St., New Haven, CT 06520 USA

**Keywords:** Substance use disorders, Incarceration, Opioids, Cocaine

## Abstract

**Background:**

The high prevalence of substance use disorders (SUDs) among incarcerated adults in the U.S. is well-known, but there has been less examination of SUD treatment and rates of incarceration among the population of adults with SUDs as the denominator. The current study uses a population-based sample to address three questions: (1) What is the rate of lifetime incarceration among the population of U.S. adults with SUDs?; (2) Among adults with SUDs, what proportion of those with incarceration histories use SUD treatment compared to those without incarceration histories?; and (3) What individual characteristics are associated with utilization of SUD treatment among adults with incarceration histories?

**Methods:**

Data were based on the National Epidemiologic Survey on Alcohol and Related Conditions-III which surveyed a nationally representative sample of U.S. adults through structured interviews. This study focused on the 10,853 respondents who had any lifetime SUD, including 2670 (weighted 22.4%) who reported a lifetime history of incarceration.

**Results:**

In the total weighted sample of respondents with SUDs, 22% had been incarcerated before but only 37% had used any alcohol use disorder treatment and 18% had used drug use disorder treatment. Controlling for confounding variables, respondents with SUDs and incarceration histories had 3.1 times the odds of using alcohol use disorder treatment and 1.6 times the odds of using drug use disorder treatment compared to their counterparts with SUDs and no incarceration histories. Having an opioid use disorder, especially heroin use disorder, and a stimulant use disorder, such as cocaine use disorder, had strong associations with any SUD treatment use.

**Conclusions:**

Many U.S. adults with SUDs have histories of incarceration but only a minority use any SUD treatment. Public health approaches that increase access and incentives to engage in and complete SUD treatment may help resolve problems of both incarceration and SUDs in the population.

Criminal justice involvement and substance abuse have long been closely linked. A systematic international review of studies reported that among male adults in prisons, 18–30% have alcohol use disorders and 10–48% have drug use disorders; among female adults in prisons, 10–24% have alcohol use disorders and 30–60% have drug use disorders [1]. In the United States, the most recent report by the Bureau of Justice Statistics estimate that 58% of adults who have been in state prisons and 63% of people who have been sentenced to jail have drug use disorders compared to 5% of the general adult population [2]. The number of incarcerated individuals has steadily increased over the past three decades; in 2016, there were 2.2 million adults incarcerated in federal and state prisons and county jail [3]. As a National Academy of Sciences report indicated [4], the rise in mass incarceration of individuals in the U.S. is partly due to drug prohibition policies implemented in the early 1970s [5], and subsequent policies such as mandatary minimum sentencing laws and new asset forfeiture rules. Thus, as substance abuse has been criminalized, there have been increasing number of individuals with substance use disorders (SUDs) being incarcerated.

Many people with SUDs in correctional facilities do not receive treatment during incarceration or post-incarceration. A recent federal report found that only 28% of people in prison and 22% of people in jail with drug use disorders participated in any drug treatment while incarcerated [2]. After incarceration, studies have found that less than half of people who had been in prison with SUDs received SUD treatment 1-year after release [6, 7]. One national study of over 70,000 people found that about 30-33% of individuals with SUD and prior-year criminal justice involvement received SUD treatment in the prior 12 months [8]. Not only was this rate much higher than the 5–7% who had received SUD treatment among those with SUDs but no prior-year criminal justice involvement, but this rate has not significantly changed from 2004 to 2014. The higher rates of SUD treatment among justice-involved individuals could be partially explained by their more frequent and severe substance use [8]. Another national study of over 8000 veterans in the Veterans Affairs Health Care for Reentry Veterans program which connects veterans exiting prison to treatment found that 57% with a SUD had at least one SUD treatment visit and 39% had three or more visits in the first year after incarceration [9]. Among veterans in the HCRV program leaving prison, being black, unmarried, homeless, and receiving disability compensation was associated with engagement in SUD treatment.

Many experts have argued for increased access to SUD treatment for people in prison or who were recently in prison [10]. Decades of research have shown that SUD treatment is effective in reducing substance use and recidivism [11–13]. In addition, studies have compared costs of SUD treatment with associated monetary benefits, and found that there was a 7:1 ratio of monetary benefits to costs, primarily due to reduced costs of crime and increased employment earnings [14]. While there are data on rates of SUDs and SUD treatment among incarcerated and formerly incarcerated adults, there are more limited national data on the population of U.S. adults with SUDs as the denominator in understanding incarceration and use of SUD treatment.

Thus, in the current study, we used a contemporary nationally representative sample of U.S. adults to answer three questions: (1) what is the incarceration rate among the population of adults with SUDs?; (2) among adults with SUDs, what proportion of those with incarceration histories use SUD treatment compared to those without incarceration histories; and (3) what factors are associated with utilization of SUD treatment among adults with incarceration histories? The descriptive results have epidemiological value for understanding this population and identifying modifiable factors related to SUD treatment that may guide development of targeted interventions. Based on past studies [8, 9] and re-entry services like the HCRV program [15] as well as a major focus from government agencies like the National Institute of Justice and the Prisoner Re-Entry Initiative on re-entry and connecting incarcerated individuals to treatment [16, 17], we hypothesized that adults with incarceration histories use SUD treatment more than those without incarceration histories. Further, we hypothesized that higher education, having an alcohol use disorder, and living in an urban area would be associated with greater likelihood of SUD treatment use.

## Methods

The National Epidemiologic Survey on Alcohol and Related Conditions-III (NESARC-III) is a cross-sectional survey of a nationally representative sample of the civilian non-institutionalized population of the United States aged 18 years or older. The sample included residents living in a variety of housing settings, including apartments, houses, gated communities, group homes, and workers’ dormitories, but did not include residents in institutions such as jail or prisons, inpatient mental hospitals, and shelters. Data for the NESARC-III was collected between April 2012 to June 2013. Multi-stage probability sampling was employed to select respondents randomly at the county, Census, and household levels. Interviewers conducted in-person structured interviews with respondents to collect information about their personal history, social activities, mental health and substance use disorders, and other health conditions. All interviewers received extensive training on field methods, received ongoing supervision, and conducted random respondent callbacks to verify data. Other details about the methodology of the NESARC-III have been detailed elsewhere [18].

Informed consent was obtained and respondents received $90 for participation. Protocols were approved by the institutional review boards at the National Institutes of Health and Westat; use of the data was approved by the institutional review board at Yale University School of Medicine.

With an overall response rate of 60.1%, the total original sample included 36,309 adult respondents. This study focused on the 10,853 respondents (29.9% of original sample) who were determined to have any lifetime SUD by a structured diagnostic interview and included 2670 (24.6%) who reported a lifetime history of incarceration and 8183 (75.4%) who reported no lifetime history of incarceration. The data were weighted through poststratification analyses to represent the U.S. civilian population based on the 2012 American Community Survey [19].

## Measures

Personal background information about respondents were collected in various domains, including demographic characteristics, finances, geographic region, military history, immigration status, and health insurance.

Lifetime incarceration was assessed with a question that asked “Since you were 18, were you ever in jail, prison, or a correctional facility?” Respondents who responded “yes” were further asked “about how long altogether were you in jail or a correctional facility since you were 18” by number of days, weeks, months, or years.

Substance use disorders were assessed with the Alcohol Use Disorder and Associated Disabilities Interview Schedule (AUDADIS-5). The AUDADIS-5 is a structured diagnostic interview developed by the National Institute of Alcohol Abuse and Alcoholism and was used to assess alcohol use disorder and specific drug use disorders, according to criteria outlined in the Diagnostic and Statistical Manual of Mental Disorders, Fifth Edition (DSM-5) [20]. The AUDADIS-5 has been extensively tested and shown to have good validity and reliability [21–23]. In this study, we used the AUDADIS-5 to examine lifetime substance use disorder diagnoses.

Lifetime homelessness was assessed with one question that asked: “Since you were 15, did you have a time that lasted at least 1 month when you had no regular place to live- like living on the street or in a car?”

The Interpersonal Support Evaluation List (ISEL) shortened 12-item version was used to measure social support [24]. Respondents were asked to rate a series of statements about emotional support (e.g., feel that there is no one to share worries and fears with) and instrumental support (e.g., would be able to find someone to help with chores if sick) on a 4-point scale from 1 (Definitely false) to 4 (Definitely true). The mean rating of these items was calculated for a social support score.

Substance abuse treatment utilization was assessed with a series of questions about any lifetime use of any self-group treatment (e.g., Alcoholics or Narcotics Anonymous meetings), detox/inpatient treatment (e.g., detoxification clinic or inpatient ward of hospital), outpatient treatment/rehabilitation (e.g., outpatient clinic, day/partial patient program, or rehabilitation program), or emergency room for alcohol and/or drug use problems.

### Data analysis

Bivariate analyses using t-tests and simple logistic regressions were conducted to compare respondents with SUDs with and without any lifetime history of incarceration on background characteristics, rates of SUDs, and SUD treatment utilization. Given the large sample sizes and the high statistical power to detect even very small differences, nearly all differences were found to be statistically significant, so effect sizes were focused on instead. For effect size measures, Cohen’s d and odds ratios (OR) with 95% confidence intervals (CIs) were calculated. Effect sizes that were small or larger were noted (d > .3 or OR < .5 or OR > 1.5) based on previous estimates of these metrics [25].

Multivariable analyses were then conducted using logistic regression analyses. First, respondents with SUDs with and without any lifetime history of incarceration were compared with one logistic regression analysis including only independent variables found to be notably different between groups in bivariate analyses (d > .3 or OR < .5 or OR > 1.5). Simultaneous entry of independent variables was used in the regression analysis. Then, two separate other logistic regression analyses were conducted to identify variables that were associated with utilization of any SUD treatment (i.e., alcohol or drug use disorder treatment). These logistic regressions were conducted separately because we theorized there would be different factors identified for each group, we had slightly different independent variables for each group, and incorporating all variables into one factorial model may be overcomplex when there is adequate statistical power for separate main effect analyses. However, in both analyses, background characteristics and SUDs were entered as independent variables; a backward stepwise entry method was used for the independent variables to retain only those variables significant at the p < .05 level. Regression analyses were conducted separately for respondents with SUDs with and without any lifetime incarceration. For all analyses, poststratification weights were applied and SAS/STAT version 9.4 was used.

## Results

Among the total NESARC sample who responded to the incarceration question (n = 36,121), a weighted 10.6% reported a history of incarceration. Among the 10,853 U.S. adult respondents with a lifetime SUD, 2670 (weighted 22.42%) reported a history of incarceration and a weighted 77.58% reported no history of incarceration. Respondents with a lifetime SUD and history of incarceration reported a mean total of 303.26 days incarcerated (sd = 15.75).

Table 1 shows bivariate differences in background characteristics between respondents with SUDs who did and did not have any history of incarceration. Among notable differences (d > .3 or OR < .5 or OR > 1.5), respondents with SUDs who had a history of incarceration were more likely to be male, to be a veteran, to have lower income, and to have been homeless than those with SUDs and no history of incarceration. Respondents with SUDs and an incarceration history were also more likely to have Medicaid and less likely to have private insurance than respondents with SUDs with no incarceration history.

**Table 1:** Background characteristics of U.S. adults with substance use disorders with and without histories of incarceration

	Substance use disorder with no history of incarceration (N = 8183)	Substance use disorder with history of incarceration (N = 2670)	Test of difference
	Mean/raw n (sd/weighted %)	Mean/raw n (sd/weighted %)	Cohen’s *d*/odds ratio (95% CI)
Background characteristics			
Age	41.29 (.24)	42.64 (.31)	.07
Sex-male	4096 (54.31)	1925 (75.64)	*2.61 (2.37, 2.88)*
Race			
White	5170 (74.96)	1475 (68.78)	.74 (.66, .83)
Black	1316 (8.29)	639 (13.83)	
Sexual orientation			
Heterosexual	7648 (95.32)	2495 (94.46)	.84 (.66, 1.07)
Gay/bisexual	455 (4.68)	152 (5.54)	
Years of education	10.33 (.05)	9.19 (.04)	− 0.28
Marital status			
Married/Live-in partner	3692 (55.86)	960 (46.57)	.69 (.54, .88)
Divorced/Separated	1522 (14.00)	753 (23.96)	
Widowed	250 (2.25)	73 (1.97)	
Never married	2719 (27.89)	884 (27.50)	
Age first married	24.32 (.10)	24.38 (.19)	.01
# of children	1.59 (.03)	2.11 (.08)	.17
Birthplace			
Foreign-born	7453 (92.47)	2522 (94.67)	.69 (.54, .88)
Native-born	729 (7.53)	146 (5.33)	
Years living in the United States	20.49 (.59)	29.14 (1.10)	.16
Urbanicity			
Urban	6869 (79.67)	2201 (77.19)	.86 (.72, 1.04)
Rural	1314 (20.33)	469 (22.81)	
Region			
Northeast	1270 (20.10)	259 (11.78)	.53 (.42, .67)
Midwest	1978 (24.52)	629 (23.88)	
South	2689 (30.56)	1043 (38.64)	
West	2246 (24.82)	739 (25.70)	
Employed full/part-time	5244 (65.05)	1266 (44.37)	.67 (.60, .76)
Ever served in the U.S. military	816 (10.67)	409 (15.84)	*1.58 (1.35, 1.84)*
Annual personal income			
$0	298 (4.07)	57 (2.05)	*2.17 (1.59, 2.98)*
$1–$9999	1409 (16.64)	662 (24.25)	
$10,000–29,999	2960 (32.96)	1122 (39.52)	
$30,000–49,999	1700 (21.04)	483 (18.13)	
$50,000–79,999	1101 (14.29)	246 (10.70)	
$80,000–99,999	297 (4.01)	39 (2.00)	
$100,000 or more	418 (6.98)	61 (3.34)	
Health coverage			
Medicare	1077 (12.94)	454 (16.24)	1.30 (1.11, 1.54)
Medicaid	1002 (9.40)	561 (17.85)	*2.09 (1.79, 2.45)*
VA/TRICARE/CHAMPUS	433 (4.90)	159 (5.96)	1.23 (.98, 1.55)
Private insurance	4860 (65.18)	989 (40.24)	*.36 (.32, .41)*
Government/state insurance	184 (2.31)	91 (3.08)	1.34 (.92, 1.97)
Any health insurance	6507 (81.86)	1823 (69.02)	*.49 (.43, .57)*
Social support score	2.56 (.003)	2.58 (.007)	.07
Any lifetime homelessness	501 (5.40)	607 (22.47)	*5.08 (4.34, 5.95)*

Reference groups are female, non-white, gay/bisexual, rural, income ≥ 100,000 participants with no history of incarceration

Italic values indicate d > .3 or OR < .5 or OR > 1.5

Table 2 details the rates of lifetime SUD diagnoses between the two groups. In bivariate comparisons, respondents with SUDs and a history of incarceration had much higher rates of almost all SUDs, but especially heroin, inhalants, hallucinogens, and cocaine than those with SUDs and no history of incarceration (all OR > 3.0). Respondents with SUDs and an incarceration history were also more likely to have used any SUD treatment for alcohol and drugs, including self-help group treatment, inpatient treatment, outpatient treatment, and emergency room services than their counterparts with no incarceration history (37% vs. 11% for any alcohol use disorder treatment, 18% vs. 4% for any drug use disorder treatment). Among respondents with alcohol use disorder and no incarceration (n = 7549), 11.5% had used any alcohol use disorder treatment compared to 39.6% who used any alcohol use disorder treatment among respondents with alcohol use disorder and a history of incarceration (n = 2386). Among respondents with drug use disorder and no incarceration (n = 2176), 14.9% had used any drug use disorder treatment compared to 31.4% who had used any drug use disorder treatment among those with drug use disorder and history of incarceration (n = 1340). Among respondents who used any SUD treatment, irrespective of incarceration history, the majority used self-help group treatment for both alcohol and drug use disorders.

**Table 2:** Rates of substance use disorders and substance use disorder treatment among U.S. adults with substance use disorders with and without histories of incarceration

	Substance use disorder with no history of incarceration (N = 8183)	Substance use disorder with history of incarceration (N = 2670)	Test of difference
	Raw n (weighted %)	Raw n (weighted %)	Odds ratio (95% CI)
Substance use disorders			
Tobacco use disorder	3693 (45.77)	1834 (71.50)	*2.97 (2.63, 3.36)*
Alcohol use disorder	7550 (92.69)	2388 (90.48)	.75 (.63, .89)
Cannabis use disorder	1429 (17.06)	798 (29.77)	*2.06 (1.77, 2.40)*
Sedative use disorder	207 (2.47)	149 (6.41)	*2.71 (2.18, 3.38)*
Heroin use disorder	57 (.74)	100 (4.17)	*5.80 (3.97, 8.48)*
Other opioid use disorder	407 (5.01)	279 (11.79)	*2.54 (2.08, 3.09)*
Cocaine use disorder	415 (5.12)	450 (16.13)	*3.56 (2.96, 4.29)*
Other stimulant use disorder	293 (3.77)	270 (11.21)	*3.22 (2.64, 3.92)*
Club drug use disorder	99 (1.10)	79 (3.07)	*2.86 (2.00, 4.08)*
Inhalant use disorder	23 (.31)	26 (1.23)	*3.99 (1.95, 8.15)*
Hallucinogen use disorder	94 (1.20)	93 (4.34)	*3.74 (2.66, 5.26)*
Any treatment for alcohol use disorder			
Self-help group treatment	795 (9.32)	882 (33.39)	*4.88 (4.26, 5.58)*
Detox/inpatient treatment	414 (4.69)	458 (16.73)	*4.09 (3.41, 4.90)*
Outpatient treatment/rehabilitation	517 (5.99)	667 (24.29)	*5.03 (4.21, 6.02)*
Emergency room	252 (3.05)	353 (13.34)	*4.90 (3.87, 6.21)*
Any alcohol use disorder treatment	926 (10.98)	977 (37.07)	*4.78 (4.17, 5.47)*
Any treatment for drug use disorder			
Self-help group treatment	295 (3.35)	438 (15.37)	*5.24 (4.37, 6.28)*
Detox/inpatient treatment	197 (2.20)	279 (9.68)	*4.76 (3.73, 6.07)*
Outpatient treatment/rehabilitation	261 (3.04)	390 (13.49)	*4.98 (4.06, 6.10)*
Emergency room	75 (.82)	136 (5.05)	*6.42 (4.63, 8.88)*
Any drug use disorder treatment	386 (4.41)	506 (17.87)	*4.72 (3.94, 5.64)*

Italic values indicate OR < .5 or OR > 1.5

Multivariable analyses were then conducted to compare respondents with SUDs with and without history of incarceration including only notable differences in bivariate analyses (d > .3 or OR < .5 or OR > 1.5). As shown in Table 3, respondents with SUDs and a history of incarceration were significantly more likely to be male, to have Medicaid coverage, and to have experienced homelessness compared to those with SUDs and no history of incarceration. Respondents also had lower income and were less likely to have private health insurance. In terms of SUDs and SUD treatment, respondents with SUDs and a history of incarceration were significantly more likely to have tobacco use, cannabis use, cocaine use, and other stimulant use disorders and more likely to have used any alcohol and drug use disorder treatment than respondents with SUDs and no history of incarceration.

**Table 3:** Multiple variable logistic regression comparing adults with substance use disorders with and without histories of incarceration

	Odds ratio (95% CI)
Background characteristics	
Sex-male	*2.86 (2.53, 3.23)*
Ever served in the U.S. military	1.16 (.95, 1.41)
Annual personal income	.94 (.89, .99)
Medicaid	1.31 (1.06, 1.62)
Private health insurance	.52 (.44, .61)
Any lifetime homelessness	*2.75 (2.29, 3.31)*
Substance use disorders	
Tobacco use disorder	*1.82 (1.59, 2.09)*
Cannabis use disorder	1.27 (1.06, 1.53)
Sedative use disorder	1.12 (.75, 1.65)
Heroin use disorder	*1.54 (.91, 2.60)*
Other opioid use disorder	1.10 (.83, 1.45)
Cocaine use disorder	*1.53 (1.16, 2.00)*
Other stimulant use disorder	*1.52 (1.15, 2.01)*
Club drug use disorder	.99 (.56, 1.75)
Inhalant use disorder	.87 (.24, 3.15)
Hallucinogen use disorder	1.34 (.83, 2.19)
Substance use disorder treatment	
Any treatment for alcohol use	*2.97 (2.653, 3.49)*
Any treatment for drug use	1.50 (1.21, 1.87)

Odds ratios represent those without histories of incarcerated, compared to those with histories of incarceration

Italic values indicate OR < .5 or OR > 1.5

To identify characteristics associated with utilization of any SUD treatment, stepwise logistic regressions were conducted on respondents with SUDs, separately for those with and without any history of incarceration (Table 4). Among respondents with SUDs and history of incarceration, having a tobacco use disorder, alcohol use disorder, heroin or other opioid use disorder, cocaine use disorder, or other stimulant use disorder were all strongly associated with any use of SUD treatment (all OR > 1.70). Notably, number of days incarcerated was not strongly associated with utilization of SUD treatment. Among respondents with SUDs and no history of incarceration, having Medicaid coverage, any lifetime homelessness, a tobacco use disorder, alcohol use disorder, heroin use, other opioid use disorder, or cocaine use disorder were all strongly associated with any use of SUD treatment (all OR > 1.70).

**Table 4:** Stepwise logistic regressions identifying factors associated with utilization of substance use disorder treatment, separately for those with and without incarceration histories

	Odds ratio (95% CI)
Adults with incarceration history	
Age	1.01 (1.00, 1.02)
Years of education	1.06 (1.01, 1.12)
Race	1.35 (1.09, 1.66)
Marital status	.70 (.57, .86)
Days incarcerated	1.00 (1.00, 1.00)
Any lifetime homelessness	1.40 (1.07, 1.83)
Tobacco use disorder	*1.71 (1.37, 2.12)*
Alcohol use disorder	*1.72 (1.16, 2.53)*
Heroin use disorder	*5.61 (2.85, 11.03)*
Other opioid use disorder	*2.08 (1.45, 2.99)*
Cocaine use disorder	*1.78 (1.30, 2.45)*
Other stimulant use disorder	*2.55 (1.75, 3.73)*
Hallucinogen use disorder	*.37 (.20, .71)*
Adults with no incarceration history	
Age	1.03 (1.02, 1.03)
Sex-male	1.20 (1.01, 1.43)
Marital status	.74 (.64, .87)
Medicaid	*1.99 (1.64, 2.43)*
Government insurance	*1.64 (1.05, 2.58)*
Any lifetime homelessness	*1.85 (1.37, 2.48)*
Tobacco use disorder	*1.99 (1.66, 2.40)*
Alcohol use disorder	*1.79 (1.34, 2.39)*
Cannabis use disorder	1.39 (1.11, 1.74)
Heroin use disorder	*7.35 (2.06, 26.17)*
Other opioid use disorder	*2.66 (1.90, 3.72)*
Cocaine use disorder	*3.37 (2.44, 4.65)*
Other stimulant use disorder	*1.57 (1.06, 2.34)*

All variables were statistically significant at the p < .05 level

Italic values indicate OR < .5 or OR > 1.5

## Discussion

Among a nationally representative sample of U.S. adults with SUDs, 22% had been incarcerated before in their lifetime, which was twice the rate found in the total NESARC sample and is consistent with the vast literature documenting the strong links between SUDs, criminal justice involvement, and incarceration [1, 2]. This finding is unique in that it reports on the proportion with incarceration histories among the population of adults with SUDs as the denominator, as most past studies have reported on the proportion of SUDs among the population of adults with incarceration histories [8, 15]. The finding also brings up questions about differences between specific SUDs and use of SUD treatment among adults with incarceration histories in relation to those without such histories. As a result, we posed three additional research questions in this study and discuss our findings below.

One research question was: What proportion of adults with incarceration histories have used SUD treatment? We found that only a minority of adults with SUDs and incarceration histories have used SUD treatment. More specifically, 37% have used any alcohol use disorder treatment and 18% had used any drug use disorder treatment. Most commonly, the type of SUD treatment that was used for both alcohol and drug use problems was self-help groups like Alcoholics Anonymous or Narcotics Anonymous, although there were also relatively high proportions who used outpatient SUD treatment. A Cochrane Review of Twelve-Step programs like Alcoholics Anonymous concluded there was lack of experimental studies demonstrating the effectiveness of these programs [26], but a more recent systematic review found that there is substantial causal evidence of their effectiveness in reducing SUDs and SUD-related outcomes [27]. These self-help SUD treatment groups have become widespread and easily accessible [28]. Perhaps, the effectiveness of these programs may not be due to its specific structure, but because they are freely available, long-term, and easy accessible [29].

A second research question was: Among adults with SUDs, do those with incarceration histories use SUD treatment more than those without incarceration histories? Our findings showed that adults with SUDs and incarceration histories were more likely to use SUD treatment than those with no incarceration histories. Those with incarceration histories had 4.8 times the odds of using alcohol use disorder treatment and 4.7 times the odds of using drug use disorder treatment as compared to those without incarceration histories. Controlling for differences in background characteristics and SUDs, those with incarceration histories still had 3.1 times the odds of using alcohol use disorder treatment and 1.6 times the odds of using drug use disorder treatment. From our data we cannot determine whether the SUD treatment was evidence-based and whether it was accessed during the incarceration period or outside of that time. However, we can say that despite those with SUDs and incarceration histories being more likely to use SUD treatment, the utilization numbers among those with SUDs overall are low, regardless of incarceration history. Thus, the larger implication of our finding is that there continue to be barriers to care for SUD treatment among people with SUDs including those with incarceration histories. This is consistent with various other studies, such as a previous national study that found only one-third of those with SUDs and criminal justice involvement in the past year used SUD treatment in the past year [8]. Some studies have shown that mental health and SUD treatment along with social services can reduce recidivism among people who were formerly incarcerated [30, 31].

Our last research question was: What individual characteristics are associated with utilization of SUD treatment among adults with incarceration histories? Our regression analyses revealed no sociodemographic factors that were strongly associated with use of SUD treatment, but having an opioid disorder or stimulant use disorder was very strongly associated with use of SUD treatment. This was true both for adults with SUDs and incarceration histories as well as those with no incarceration histories and characteristics associated with SUD treatment utilization were largely similar between the two groups. Thus, it seems SUDs involving the “hard drugs” was more associated with SUD treatment than the more prevalent SUDs involving alcohol or cannabis. This finding is entirely consistent with a previous study that examined multiple international epidemiological surveys including the U.S. National Comorbidity Survey and found that cocaine and heroin use significantly predicted SUD treatment-seeking behaviors in the general population [32]. We agree with the study authors’ interpretation that this finding may be due to the possibility that opioids and stimulants are more likely than other substance to lead to impairments or symptoms that promote treatment seeking and often occurs later in the progression of drug use after other “gateway drugs” like alcohol and cannabis. This may also be important in the context of a recent study that found increased criminal justice involvement with increased opioid use [33]. Moreover, there is cause for concern that there is wide variability in quality, type, and intensity of treatment particularly for opioid use disorder. For example, one national study found that only 4.6% of justice-referred clients with opioid use disorder received agonist treatment compared to 40.9% of those referred from some other entity although agonist treatment can be highly effective for opioid use disorder [34]. This has spurred programs like the ones launched by the Rhode Island Department of Corrections [35] and by Rikers Island in New York City [36] to provide medication-assisted treatments for people with opioid use disorder in correctional facilities.

Together, the findings of this study highlight the need for public health interventions to address the high rates of SUDs among U.S. adults who have been involved in the criminal justice system. While we found higher utilization rates of SUD treatment among those with incarceration histories including those who have problems with opioids and stimulants, there is still much opportunity for increasing treatment utilization and ensuring use of evidenced-based practices. Importantly, a focus on prevention of SUDs would reduce the numbers who need SUD treatment and may possible curb criminal justice involvement [37, 38]. At the same time, the high incarceration rate among those with SUDs is not simply due to substance abuse, but a host of social determinants, such as unstable housing, poverty, and social networks; in fact, SUDs and criminal justice involvement share many of these same risk factors [39, 40]. Thus, comprehensive models of care that address these factors should be encouraged and evaluated for their effectiveness at population-based levels.

Our study had several limitations of note. First, this was a large-scale epidemiological examination of associations and lifetime incarceration and SUDs were assessed. The directionality of associations cannot be determined and it is likely many associations we found are bi-directional [41]. For example, we do now know whether the SUD treatment occurred before, during, and/or after incarceration. Second, the data were based on respondent self-report and reports about substance use and histories of incarceration may have been subject to various response biases. Third, the NESARC-III only sampled non-institutionalized adults so adults who are currently incarcerated or hospitalized were not included and so we may have missed an important segment of the population for our study. Fourth, we did not have detailed data on SUD treatment utilization so information about the intensity of SUD treatment services received and length of time were missing, which would be important for future study. These limitations notwithstanding, we believe the study provides important population data on SUDs and SUD treatment in the U.S. and our focus on adults with SUDs and incarceration histories highlights the need for more public health approaches to increase SUD treatment utilization in this population.

## Conclusions

The strong association between SUDs and incarceration in the U.S. adult population suggests it is important to increase access and incentives to access SUD treatment during and after incarceration. This epidemiological study provides contemporary data on national rates of SUD treatment utilization among adults with SUDs and incarceration histories. While adults with SUDs and incarceration histories reported greater utilization of SUD treatment, there were generally low SUD treatment utilization rates overall regardless of incarceration history. These results underscore the importance of increasing access to and motivation for SUD treatment, and there may be both common and different barriers for individuals with SUDs depending on incarceration history. Greater need for prevention and intervention for the nexus between criminal justice and substance use problems is needed.
